# Low-intensity pulsed ultrasound enhances callus consolidation in distraction osteogenesis of the tibia by the technique of lengthening over the nail procedure

**DOI:** 10.1186/s12891-019-2490-7

**Published:** 2019-03-14

**Authors:** Mi Hyun Song, Tae-Jin Kim, Sung Hyun Kang, Hae-Ryong Song

**Affiliations:** Department of Orthopaedic Surgery and Institute for Rare Diseases, Korea University Medical Center, Guro Hospital, 148 Gurodong-ro, Guro-gu, Seoul, 08308 South Korea

**Keywords:** Distraction osteogenesis, Low-intensity pulsed ultrasound, Healing index, Callus maturation

## Abstract

**Background:**

Low-intensity pulsed ultrasound (LIPUS) has been widely accepted in promoting the fracture healing process. However, there have been limited clinical trials focused on the efficacy of LIPUS during distraction osteogenesis (DO) by the technique of lengthening over the nail procedure. The purpose of the current study was to evaluate the efficacy of LIPUS during DO.

**Methods:**

We retrospectively evaluated 30 patients (60 segments) who underwent simultaneous bilateral tibial lengthening over the nail. The patients were grouped into the LIPUS group and the control group based on LIPUS stimulation. The two patient groups were compared for demographic data (sex, age at operation, preoperative height, BMI, and smoking history), qualitative assessments of the callus (callus shape and type), external fixation index, and four cortical healing indexes.

**Results:**

Fifteen patients (30 segments) were classified as the LIPUS group, and another 15 patients (30 segments) were classified as the control group. No significant differences were found in the assessed demographic data between the groups. LIPUS stimulated a more cylindrical, more homogenous, and denser type of callus formation at the end of the distraction phase. The two groups exhibited equivalent outcomes in terms of external fixation index (*p* = 0.579). However, significant differences were found in healing indexes of the anterior and medial cortices (*p* <  0.001 and *p* = 0.002, respectively). The healing indexes of those cortices in the LIPUS group (mean of 36.6 days/cm and 32.5 days/cm, respectively) reflected their significantly faster healing compared to the control group (mean HI of 57.5 days/cm and 44.2 days/cm, respectively). There were no LIPUS-related complications.

**Conclusions:**

LIPUS is a noninvasive and effective adjuvant therapy to enhance callus maturation during DO. It enhances callus consolidation and may have a positive effect on the appropriate callus shape and type.

## Background

Distraction osteogenesis (DO) using an external fixator has been successfully used in limb lengthening for short stature or limb length discrepancy. The consolidation of regenerated callus during DO can be affected by various factors including host factors (age and underlying disease), local factors (adjacent soft tissue condition and concomitant infection), and surgical factors (surgical technique, stability of the frame, and modulation of the distraction rhythm) [1–4]. Long-term external fixation, related to delayed consolidation, may lead to complications such as pin site infection and adjacent joint contracture, as well as subsequent socioeconomic and psychological burdens on the patient [3, 5].

To reduce these complications and burdens on the patient, several techniques have been employed to enhance callus maturation. Local application of autogenous and allogenous iliac bone, biphasic calcium phosphate [6], platelet-rich plasma (PRP) [7, 8], and demineralized bone matrix (DBM) [9] have been used to induce biological stimulation of callus consolidation. Furthermore, systemic application of parathyroid hormone [10], growth hormone [11], and bisphosphonate [12] have been investigated. However, further investigation of the optimal dose and potential risk of complications of these biological materials is required for their wide clinical use.

Low-intensity pulsed ultrasound (LIPUS), which has been widely accepted for promoting the fracture healing process [13–17], may be another option for adjuvant therapy to accelerate consolidation of the regenerated callus. Although there have been several in vivo studies evaluating the efficacy of LIPUS on callus maturation during DO [18–23], there have been limited clinical trials focused on the efficacy of LIPUS during DO [24–26]. Therefore, this clinical investigation was performed to evaluate the efficacy of LIPUS during DO under the hypothesis that LIPUS would enhance callus maturation.

## Methods

The medical and radiographic records of patients who underwent tibial lengthening in our institute from October 2009 to October 2015 were reviewed. Only patients who were skeletally mature, underwent simultaneous bilateral tibial lengthening over the nail, and had no medical illness (such as bone metabolic disorders or neuromuscular disorders) or history of trauma were included. Patients who developed an infection (including pin-site infection and acute osteomyelitis) during DO, received adjuvant therapy other than LIPUS, exhibited a lack of compliance, or had incomplete medical and radiographic records were excluded. A total of 30 patients (60 tibiae) were enrolled in this study. The mean age at tibial lengthening was 22.1 years (17.5–34.0), and the patients were followed for an average of 5.6 years (1.7–8.2).

### Surgical procedure

The tibial lengthening over the nail procedure was performed by the senior author (HRS) using an Ilizarov apparatus (U & I Co. Ltd., Seoul, Korea) and AO unreamed tibial nails. At the mid-diaphyseal level, a transverse osteotomy was performed using the multiple-drill-hole method. At the time of osteotomy, the nail was concomitantly inserted and reached the distal metaphysis as the development of a valgus or varus deformity was monitored. Two proximal interlocking screws were inserted in the medio-lateral direction. The preconstructed Ilizarov apparatus was then mounted to the tibia. Range of motion exercises for the adjacent joints and full weight bearing were started on postoperative day 3. After 7–10 days of consolidation, tibial lengthening was initiated at a rate of 0.25 mm every 6 h. The actual distraction rhythm was determined depending on the morphological features of the callus in each case [27]. After achieving the desired length, the intramedullary nail was interlocked. The external fixator was removed when we observed a bridging callus at 2 of 4 cortices on plain radiographs. A short leg cast was used for the next 2 weeks to prevent a regenerate fracture, and a brace was then applied until complete consolidation of at least 3 of 4 cortices. The patients were followed every week during the first month, every 2 weeks during the distraction phase, and then monthly during the consolidation phase.

### LIPUS protocol

LIPUS stimulation was initiated during the distraction phase and maintained until the early consolidation phase. It was performed over the corticotomy site and the distraction gap for 20 min a day. A well-trained physician carried out the LIPUS and instructed the patient on the procedure during the hospitalization, and the patients performed self-treatment after discharge. The compliance of each patient was confirmed in the out-patient clinic by assessing the remaining usage of the device. The Sonic Accelerated Fracture Healing System (Exogen Inc., Piscataway, NJ, USA) and BH-1000 ultrasound bone healing device (Orthoheal, Seoul, Korea) were used for the LIPUS. The characteristics of the LIPUS signal were a frequency of 1.5 MHz, a signal repetition rate of 1 kHz, a pulse width of 200 μs, 117 mW of power, and an intensity of 30 mW/cm^2^ [26, 28].

### Evaluations

The patients were grouped into the LIPUS group and the control group based on LIPUS. To investigate the efficacy of LIPUS during DO, the two patient groups were compared for demographic data (sex, age at operation, preoperative height, BMI, and smoking history), qualitative assessments of the callus [27], external fixation index, and four cortical healing indexes.

Radiographic evaluations were performed using standard tibia/fibula anteroposterior and lateral radiographs and a slit scanogram (as described by Bell and Thompson) [29] both before tibial lengthening and at every postoperative visit at the out-patient clinic. Initial tibial length, amount of lengthening, and lengthening percentage were assessed. The callus shape and type according to Li et al. [27] were assessed for qualitative assessments of the callus. The callus shape was classified as fusiform, cylindrical, concave, lateral, or central. The callus type was classified as normal-, intermediate-, or low-density. These parameters were investigated twice, at the end of the distraction phase and at the time of external fixator removal, to observe the progression of callus maturation. The external fixation index (EFI) was calculated as the duration of external fixation in days divided by the length gained in cm, and the external fixator was removed when a bridging callus was observed at 2 of 4 cortices. In comparison, the healing index (HI) was calculated as the duration of complete consolidation in days divided by the length gained in cm, and complete consolidation was defined as formation of cortical bridging at at least 3 of 4 cortices.

Additionally, complications other than infection (including pin-site infection and acute osteomyelitis) were assessed [3].

### Statistical analysis

We used the Mann-Whitney test for continuous data not following a normal distribution and the independent t-test for continuous data following a normal distribution. Categorical data were analyzed using Fisher’s exact test. Statistical analyses were performed using SPSS software, version 20.0 (SPSS, IBM Corp., Chicago, IL). *P*-values < 0.05 were regarded as statistically significant.

## Results

**Fig. 1:**
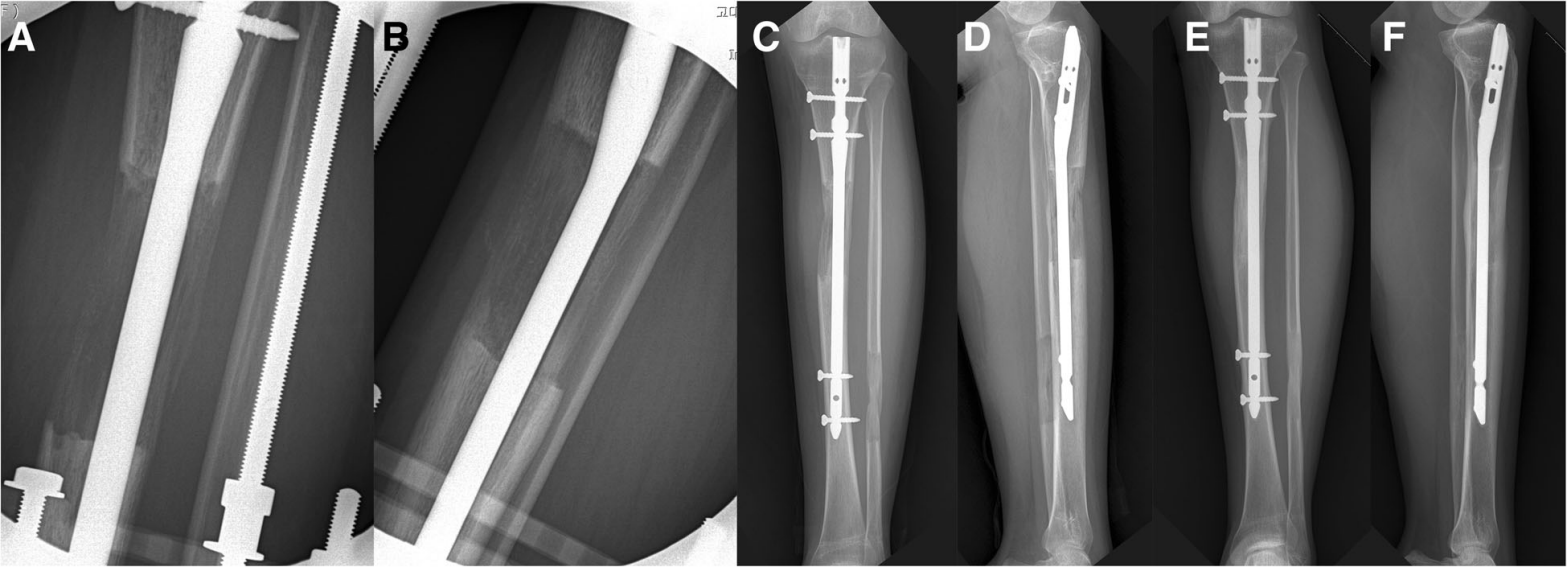
Sequential radiographic images of the left tibia in a 22-year-old female patient who received low-intensity pulsed ultrasound (LIPUS) after 7.3 cm of tibial lengthening. At the end of the distraction phase, the anteroposterior (AP) (**a**) and lateral (**b**) images show a callus with a cylindrical shape and intermediate density. At the time of external fixator removal, the AP (**c**) and lateral (**d**) images show progression of callus consolidation with a cylindrical shape and normal density. The external fixation index of the patient was 29.6 days/cm. At 9 months postoperation, the AP (**e**) and lateral (**f**) images show full corticalization of the regenerated callus

**Fig. 2:**
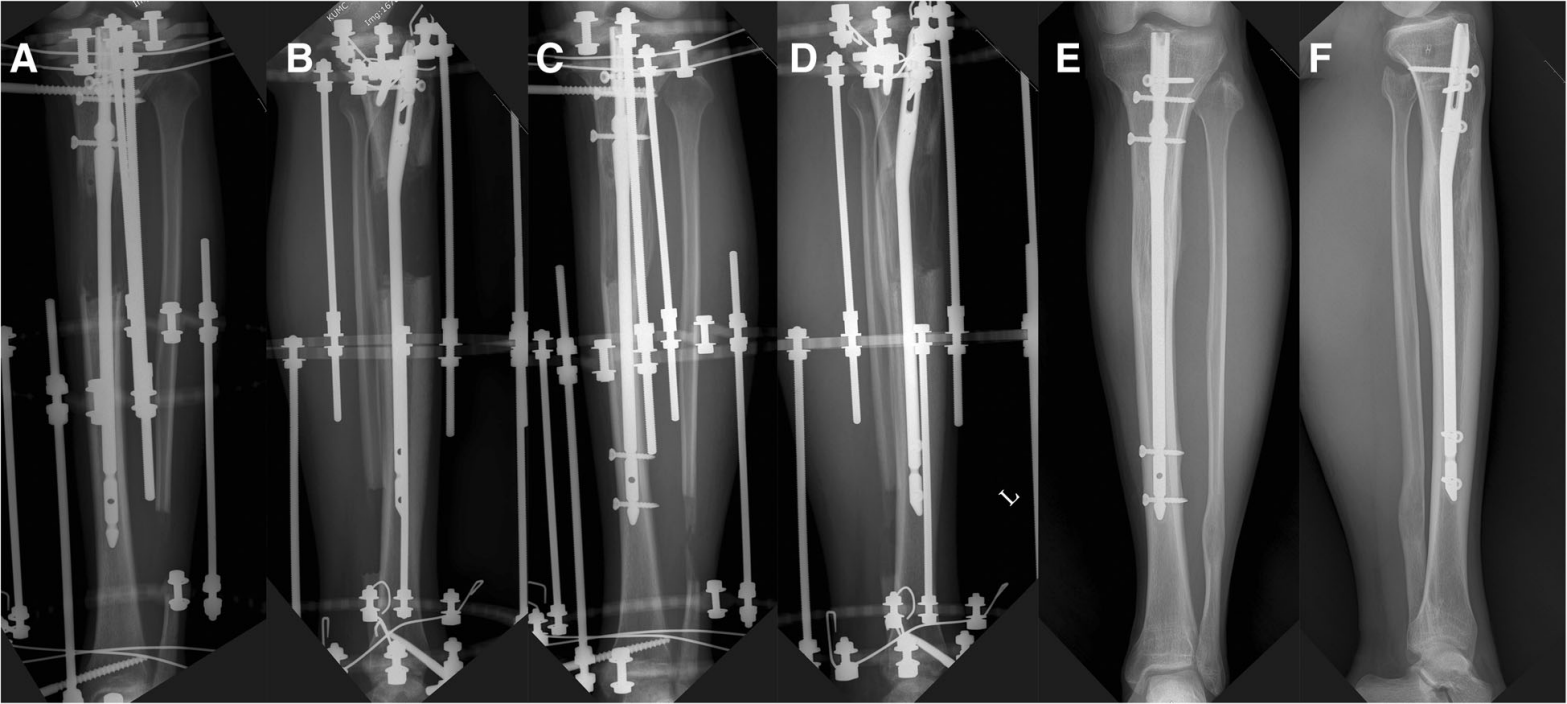
Sequential radiographic images of the left tibia in a 19-year-old female patient without low-intensity pulsed ultrasound (LIPUS) stimulation after 8.1 cm of tibial lengthening. At the end of the distraction phase, the anteroposterior (AP) (**a**) and lateral (**b**) images show a callus with a cylindrical shape and low density. At the time of external fixator removal, the AP (**c**) and lateral (**d**) images show progression of callus consolidation with a cylindrical shape and intermediate density. The external fixation index of the patient was 29.4 days/cm. At 17 months postoperation, the AP (**e**) and lateral (**f**) images show full corticalization of the regenerated callus

Fifteen patients (30 tibial segments) were classified as the LIPUS group (Fig. 1), and another 15 patients (30 tibial segments) were classified as the control group (Fig. 2). The demographic data of the LIPUS and control groups are presented in Table 1. No significant differences between the groups were found for the assessed demographic data including sex, age at operation, preoperative height, BMI, and smoking history. In addition, no significant differences were found between the groups in terms of the initial tibial length and amount and percentage of tibial lengthening (Table 2).

**Table 1:** Demographic data of patients undergoing bilateral tibial lengthening

Variable	LIPUS group	Control group	*P*-value
Number of patients	15	15	
Number of tibial segments	30	30	
Sex (M:F) (number of tibia)	24:6	25:5	1.000†
Age at operation (years)*	22.1 (17.5 to 34.0)	20.6 (17.9 to 25.4)	0.155‡
Preoperative height (cm)*	165.4 (153 to 174)	163.2 (147 to 173)	0.073‡
BMI (kg/m^2^)*	21.7 (17.2 to 26.2)	21.8 (17.4 to 27.8)	0.941‡
Smoking history (smoker: nonsmoker) (number of tibia)	2:28	4:26	0.671†
Follow-up (years)*	5.3 (1.7 to 8.2)	5.8 (3.9 to 7.8)	0.790‡

LIPUS, Low-intensity pulsed ultrasound

*Values are expressed as mean, with range in parentheses

† Fisher’s exact test, ‡ Mann-Whitney test

**Table 2:** Radiographic comparison between the LIPUS and control groups

Classification	LIPUS group (*N* = 30)	Control group (*N* = 30)	*P*-value†
Initial tibial length (mm)*	318 (262 to 353)	313 (258 to 360)	0.383
Amount of lengthening (mm)*	82 (70 to 105)	76 (50 to 100)	0.065
Lengthening percentage (%)*	25.8 (20.3 to 36.6)	24.7 (13.9 to 35.5)	0.350
External fixator index (days/cm)*	29.4 (16.5 to 44.8)	29.6 (17.3 to 45.8)	0.579
Healing index (days/cm)*			
Anterior cortex	36.6 (21.7 to 51.6)	57.5 (32.5 to 64.8)	**< 0.001**
Posterior cortex	24.3 (15.3 to 43.0)	25.9 (16.9 to 44.8)	0.367
Medial cortex	32.5 (19.5 to 47.6)	44.2 (29.3 to 53.4)	**0.002**
Lateral cortex	29.4 (16.5 to 44.8)	29.6 (17.3 to 45.8)	0.579

LIPUS, Low-intensity pulsed ultrasound

* Values are expressed as mean, with range in parentheses

† Independent t-test

The callus shape and type, classified according to Li et al. [27], for each treatment group are presented in Table 3. At the end of the distraction phase, there were significant differences in callus shape and type between the groups (*p* = 0.005 and *p* <  0.001, respectively). Calluses of the LIPUS group were more cylindrical, more homogenous, and denser type than those of the control group. At the time of external fixator removal, however, no significant differences were observed between the groups in callus shape and type (*p* = 0.055 and *p* = 0.313, respectively).

**Table 3:** Comparison of callus shape and type according to Li et al. [27] between the two groups

Classification	At the end of distraction phase			At the time of ex-fix removal		
	LIPUS group (*N* = 30)	Control group (*N* = 30)	P-value*	LIPUS group (*N* = 30)	Control group (*N* = 30)	*P*-value*
Callus shape			**0.005**			0.055
Fusiform	0	0		4	3	
Cylindrical	19	13		23	15	
Concave	11	8		3	10	
Lateral	0	9		0	2	
Central	0	0		0	0	
Callus type			**< 0.001**			0.313
Normal-density	0	0		30	29	
Intermediate-density	24	4		0	1	
Low-density	6	26		0	0	

LIPUS, Low-intensity pulsed ultrasound

* Fisher’s exact test

There was no significant difference in the EFI between the groups. However, significant differences were found in the HIs of the anterior and medial cortices (*p* <  0.001 and *p* = 0.002, respectively) (Table 2). The HIs of those cortices in the LIPUS group (mean of 36.6 days/cm and 32.5 days/cm, respectively) reflected their significantly faster healing compared to the control group (mean HI of 57.5 days/cm and 44.2 days/cm, respectively).

Six complications were encountered in the LIPUS group, whereas five complications occurred in the control group (Table 4). Equinus deformity was the most common complication in both groups and was managed using a Vulpius procedure with or without gradual distraction through application of an additional foot frame. One case of impending compartment syndrome developed in the LIPUS group, and the patient required a fasciotomy at 1 day postoperation. In addition, valgus angulation of the tibia was seen in one patient in the control group. The patient refused to undergo any intervention to treat the deformity, and the deformity persisted without any further symptoms. No LIPUS-related complications developed in the LIPUS group.

**Table 4:** Comparison of complications between the two groups

Complication	LIPUS group (*N* = 30)	Control group (*N* = 30)	*P*-value*
Ankle equinus	5	4	0.739
Impending compartment syndrome	1	0	
Peroneal nerve irritation	0	0	
Fibula related	0	1	
Regenerate fracture	0	0	
Nonunion	0	0	
LIPUS-related	0		

LIPUS, Low-intensity pulsed ultrasound

* Fisher’s exact test

## Discussion

We performed a clinical investigation on the efficacy of LIPUS during DO. The findings of the current study showed that LIPUS stimulated a more cylindrical, more homogenous, and denser type of callus formation in the early stage of DO (at the end of distraction phase) and promoted faster callus consolidation, especially in the anterior and medial cortices.

LIPUS is a mechanical stimulation method using high-frequency acoustic pressure waves. It is speculated that LIPUS exerts low-magnitude mechanical stress at the application site and acts through a functional loading effect [30]. This may provoke a cascade of biological responses during cellular reaction processes in the inflammation, angiogenesis, chondrogenesis, and endochondral and intramembranous ossification stages, as well as the bone remodeling period [18, 30]. Accordingly, LIPUS stimulates differentiation and proliferation of osteogenic cells, activation of osteoblasts, and synthesis of osteocalcin [23]. Expression of messenger ribonucleic acid (mRNA) and synthesis of fibroblast growth factor (FGF), vascular endothelial growth factor (VEGF), platelet-derived growth factor (PDGF), and prostaglandin E2 are also increased via LIPUS stimulation [17, 31–34].

To produce acceptable outcomes with LIPUS therapy, a well-formulated LIPUS protocol is required. The LIPUS protocol established in previous studies is a frequency of 1.5 MHz, a signal repetition rate of 1 kHz, a pulse width of 200 μs, and an intensity of 30 mW/cm2 with a 20-min application time per day [23, 26, 28, 35]. Because these previous studies achieved satisfactory outcomes via LIPUS during either DO or fracture healing, this study was conducted using the same LIPUS protocol: the characteristics of the LIPUS signal were a frequency of 1.5 MHz, a signal repetition rate of 1 kHz, a pulse width of 200 μs, 117 mW of power, and an intensity of 30 mW/cm^2^.

In this study, LIPUS stimulation was initiated during the distraction phase; however, the timing for application of LIPUS during DO is not standardized [26]. Most studies have preferred to conduct LIPUS during the consolidation phase [18, 19, 21, 23, 24, 36]. However, we hypothesized that LIPUS could play an important role in enhancing callus consolidation when applied during the distraction phase because active angiogenesis, which is closely related to mineralization in the distraction gap, occurs during the distraction phase [37]. Aronson demonstrated that regional blood flow at the corticotomy site increases 10-fold during the early distraction phase [38]. This increased blood flow decreases after surgery but remains up to 3 times greater than normal until 17 weeks postoperation. The increase in blood flow in this phase leads to an increase in hematopoietic function, differentiation of pluripotential cells, and active angiogenesis [37].

The efficacy of LIPUS is debatable. Recently, Simpson et al. [39] demonstrated that callus consolidation was not significantly stimulated by LIPUS therapy but was stimulated by smoking status. A meta-analysis including the mentioned study [40] concluded that LIPUS influenced neither treatment time nor decrease in risk of complications. However, several clinical trials have suggested the effectiveness of LIPUS on DO (Table 5). El-Mowafi and Mohsen [24] demonstrated that LIPUS induced a 38% significant reduction in the HI using the conventional Ilizarov technique. Salem and Schmelz [26] found that LIPUS reduced the HI by 27% in posttraumatic tibial defects after comminuted diaphyseal tibial fractures. Dudda et al. also concluded that the EFI could be shortened in patients who receive LIPUS therapy (32.8 days/cm in the LIPUS group and 44.6 days/cm in the control group), although their data did not achieve statistical significance [25]. In addition, Tsumaki et al. found that LIPUS therapy significantly increased bone mineral density, accelerates callus maturation, and reduced the duration of external fixation application [41]. Concurrent with these previous studies, the current study also indicated that LIPUS improved the quality of the callus with a more cylindrical, more homogenous, and denser type of callus formation and led to faster callus consolidation, especially in the anterior and medial cortices. In addition, no LIPUS-related complications developed among the LIPUS group during the study.

**Table 5:** Healing index in previous comparative studies demonstrating the efficacy of LIPUS stimulation during tibial lengthening

Article	Type of external fixation	Gain of length (cm)	Timing of LIPUS	Treatment time	Group	Total no. of segments	Age at operation (yrs)	Healing index	
								(d/cm)	*P* value
El-Mowafi and Mohsen [24]	Ilizarov	6.1 (5 to 8)	Consolidation period	20 min/day	LIPUS	10	35 (18 to 45)	30 (27 to 36)	< 0.001
					control	9		48 (42 to 75)	
Dudda et al. [25]†	Regazzoni (23) Ilizarov (6) Hybrid type (7)	6.6 (2.5 to 14.0)	–	20 min/day	LIPUS	16	34.9 (17 to 64)	32.8 ± 13.1	0.116
					control	20	42.4 (16 to 69)	44.6 ± 26.8	
Salem and Schmelz [26]	Ilizarov	7.9	Distraction and consolidation period	20 min/day	LIPUS	12	32	33	–
					control	9	29	45	
Current study‡	Lengthening over nail	7.9 (5.0 to 10.5)	Distraction and early consolidation period	20 min/day	LIPUS	30	22.1 (17 to 34)	36.6 (21.7 to 51.6)	< 0.001
					control	30	20.6 (17 to 25)	57.5 (32.5 to 64.8)	

*LIPUS*, Low-intensity pulsed ultrasound

* Data presented in the parenthesis mean range

† The authors measured external fixation index instead of healing index

‡ Healing index at the last consolidated cortex among the four cortices

This study demonstrated that LIPUS enhanced callus consolidation, especially in sites with a thin covering of soft tissue (anterior and medial cortices). It is known that inadequate soft tissue coverage is closely related to late consolidation of the regenerated callus during DO; hence, calluses on these two cortices have a higher risk of delayed consolidation than those of the posterior and lateral cortices [7]. However, a thinner covering of soft tissue causes less attenuation of the LIPUS signal [23], which may increase the efficacy of LIPUS stimulation on the anterior and medial cortices during DO.

However, in terms of EFI, no significant positive effect was observed by LIPUS stimulation. We supposed that this finding was related to the lengthening over the nail procedure used in the current study. It is possible to remove the external fixator after achieving consolidation of only 2 of 4 cortices using the lengthening over the nail procedure, while it is safe to remove the external fixator after achieving complete consolidation of at least 3 of 4 cortices using the conventional Ilizarov procedure.

This study was limited by its retrospective nature. Also, the number of patients opting for LIPUS was not large enough due to the added cost of the therapy, limiting the number of patients recruited. These factors could affect the statistical significance. Nevertheless, all patients in this study were surgically treated by a single surgeon at a single institution with the same standardized LIPUS protocol. In addition, to the best of our knowledge, this is the first study evaluating the efficacy of LIPUS during DO by the technique of lengthening over the nail procedure.

## Conclusions

LIPUS is a noninvasive and effective adjuvant therapy to enhance callus maturation during DO. It also enhances callus consolidation and may have a positive effect on appropriate callus shape and type.

## Abbreviations

DBM: Demineralized bone matrix
DO: Distraction osteogenesis
EFI: External fixation index
FGF: Fibroblast growth factor
HI: Healing index
LIPUS: Low-intensity pulsed ultrasound
PDGF: Platelet-derived growth factor
PRP: Platelet-rich plasma
RNA: Ribonucleic acid
VEGF: Vascular endothelial growth factor
